# Practice in Information Technology Support for Fangcang Shelter Hospital during COVID-19 Epidemic in Wuhan, China

**DOI:** 10.1007/s10916-021-01721-y

**Published:** 2021-02-19

**Authors:** Qian He, Hui Xiao, Han-ming Li, Bei-bei Zhang, Cheng-wei Li, Fang-jian Yuan, Sha-sha Yu, Fang Zhang, Ping Kong

**Affiliations:** Information Center, Zhongnan Hospital of Wuhan University, Wuhan, 430071 China

**Keywords:** COVID-19, Fangcang shelter hospital, IT infrastructure, Information system, Nosocomial infection

## Abstract

In confronting the sudden epidemic of COVID-19, China and other countries have been under great deal of pressure to block virus transmission and reduce death cases. Fangcang shelter hospital, which is converted from large-scale public venue, is proposed and proven to be an effective way for administering medical care and social isolation. This paper presents the practice in information technology support for a Fangcang shelter hospital in Wuhan, China. The experiences include the deployment strategy of IT infrastructure, the redesign of function modules in the hospital information system (HIS), equipment maintenance and medical staff training. The deployment strategy and HIS modules have ensured smoothness and efficiency of clinical work. The team established a quick response mechanism and adhered to the principle of nosocomial infection control. Deployment of network and modification of HIS was finished in the 48 hours before patient admittance. A repair hotline and remote support for equipment and software were available whenever medical workers met with any questions. No engineer ever entered the contaminated areas and no one was infected by the coronavirus during the hospital operation. Up to now, Fangcang shelter hospital is adopted by many regions around the world facing the collapse of their medical systems. This valuable experience in informatization construction and service in Wuhan may help participators involving in Fangcang shelter hospital get better information technology support, and find more practical interventions to fight the epidemic.

## Introduction

In December 2019, the coronavirus disease 2019 (COVID-19) caused by Severe Acute Respiratory Syndrome Coronavirus 2 (SARS-CoV-2) broke out in Wuhan, Hubei province of China [1]. Over the next few weeks, researchers found human-to-human transmission cases within the community [2] and gained a deeper understanding of the diseases’ characteristics [3, 4]. The Chinese central government decisively vowed to take preventive and control measures of category A infectious disease to fight against the pneumonia around the country [5]. Social distancing and epidemic control policy package was enacted to reduce the spread of the virus [6]. Medical authorities in Wuhan quickly assigned several batches of designated hospitals for the epidemic, comprising fever clinics, isolation wards and intensive care units. However, confirmed cases increased rapidly and consequently caused difficulties with hospitalization since the beginning of February, 2020. Wuhan Municipal Headquarters for COVID-19 Prevention and Control subsequently began to convert large-scale public spaces, such as exhibition halls, gymnasiums and workshops, into Fangcang shelter hospitals. The Fangcang shelter hospital is a temporary point of care that has basic functions of isolation, triage, basic medical care, frequent monitoring and rapid referral, and essential living and social engagement [7]. A total of 16 Fangcang hospitals were put into use in Wuhan, receiving more than 12,000 non-critically ill patients with COVID-19 until March 10, 2020 [8]. And the non-pharmaceutical interventions from 2 February, including centralized isolation and quarantine of all patients, have reduced the total infections in Wuhan by 69.6% as of 8th March 2020 [9].

Fangcang shelter hospitals have several unique characteristics, including strict nosocomial infection control, rapid construction, massive scale and decentralized human resources [7]. Thus, patient management, treatment, drug supply, equipment maintenance and user training in Fangcang hospital differ from those in a traditional hospital. To adapt these characteristics, the information technology department (IT Dept.) and hospital information system (HIS) had to make many changes. Wuhan Living Room Fangcang Shelter Hospital, the biggest one in Wuhan with 2000 beds, was managed by a team from Zhongnan Hospital of Wuhan University [10]. IT Dept. of the team was responsible for informatization construction and service. The IT Dept. supported the hospital operation smoothly between its opening and suspension. During the period, deployment strategy of network and server, process optimization in HIS, maintenance, training and some other responsibilities of the IT Dept. were explored and improved for the makeshift hospital.

In subsequent months, COVID-19 has posed a great threat to global health. Most countries and regions around the world encountered challenges, and many of them faced the daunting task of treating an overwhelmingly large number of coronavirus-infected patients. And a second COVID-19 epidemic wave seemed to be settling in Europe and the rest of the World since autumn [11, 12].The World Health Organization has characterized COVID-19 as a pandemic on 11th March 2020 and suggested other nations learn from China in the fight against the disease [13]. Many regions adopted Fangcang hospitals as alternatives to crowded traditional hospitals in peak period of the pandemic. The experience in IT construction and operation in China may greatly help IT engineers and medical workers in these regions. Thus, the work of IT Dept. in Wuhan Living Room Fangcang Shelter Hospital is shared in this paper with the international medical informatization community.

## Deployment strategy of IT infrastructure

There were two deployment strategies available for IT infrastructure, including server and network, in Fangcang shelter hospitals. One was independent, another one was dependent. An independent deployment made a Fangcang shelter look like an independent hospital that was equipped with exclusive IT infrastructure. The dependent deployment made Fangcang shelter look like a remote department of a host hospital. It shared the server of host hospital rather than deploying its own infrastructure. Terminal equipment in Fangcang shelter accessed the server in the host hospital via a local area network (LAN). After a detailed comparative analysis, the dependent deployment was determined to be more fitting for Fangcang shelter in China (Table 1). There were several reasons for this decision. First, every Fangcang shelter was administered by a large Grade 3A hospital according to the government plan. The larger hospital could bring its mature human resources, regulatory regime and hospital infection control experience into Fangcang shelter, as well as informatization construction and service. Second, Fangcang shelter hospitals were ordered to finish construction in one or two days with low cost [7]. Only dependent deployment would match this requirement regarding speed and cost.

**Table 1:** Deployment strategy of IT infrastructure in Fangcang shelter hospital

**Strategy**	**Description**	**Maintenance**	**Data management**	**Time consumption**	**Expenditure**
Independent	New server, database, application and configuration	Engineers can deal with problems after learning the new configuration	New Data organization and processor	Longer	Higher
Dependent	Additional department deployed on existent IT infrastructure	Local engineers who were already familiar with configuration can deal with various problems immediately	Uniform data structure and processor	Shorter	Lower

In the dependent deployment strategy, LAN between the Fangcang shelter hospital and its host hospital was the focus of construction. Zhongnan Hospital and the Wuhan division of China Telecom Group Co., Ltd. constructed a high speed virtual private network (VPN) tunnel between the two sites. Inside Fangcang shelter, wireline local area network (WLAN) connected all of the terminal equipment to wireless routers and then to servers over VPN. All data generated in Fangcang shelter was stored, processed, analysed, extracted and reported by facilities and engineers in the host hospital. Taking data protection into consideration, the IT Dept. stepped up security at network ports by adjusting intranet settings in the router and firewall. Engineers also encouraged all customers to protect themselves against potential risks by avoiding clicking on suspicious links and e-mails. Additionally, heightened security was set on servers and terminals.

## Function module of HIS

The HIS in the Fangcang shelter hospital was redesigned and modified in some respects to adapt to the unique medical environment. It followed a simplified hospital admissions process and a complex referral procedure. Unlike the free-write style of medical records in general hospitals, most medical documents provided templates that medical workers could fill out, reducing the amount of writing and improving consistency. A mobile electronic medical record system with a user-friendly interface, such as selecting icons or buttons, was available for doctors. Laboratory tests, imaging examinations and pharmacy services were integrated in HIS to varying extents. Thus, all data involving diagnosis and treatment were generated from, stored in, and managed by HIS. A dozen statistical reports were directly extracted from HIS to support managers making decisions both inside and outside of the hospital. The main functions of the system that were customized are introduced below.

### Patient admittance and referral

The admittance in Fangcang shelter hospital followed a unique process. Most patients came from community isolation sites, whose admission applications were sent to Fangcang shelter hospital headquarters in pre-defined tabular form before their set out. If he arrived at the ward and conform to the admittance criteria of Fangcang shelter [10], the registration and bed allocation would be completed in HIS for him. HIS provided two ways for patient registration after admittance—spreadsheet import and manual input. In order to reduction personnel gathering, the registration was done at nurse station near the reserved bed, rather than at an admission registration center. All functions relating to charge and settlement between patient and hospital in HIS were cancelled, due to the free treatment policy issued by Chinese government [14].

**Fig. 1:**
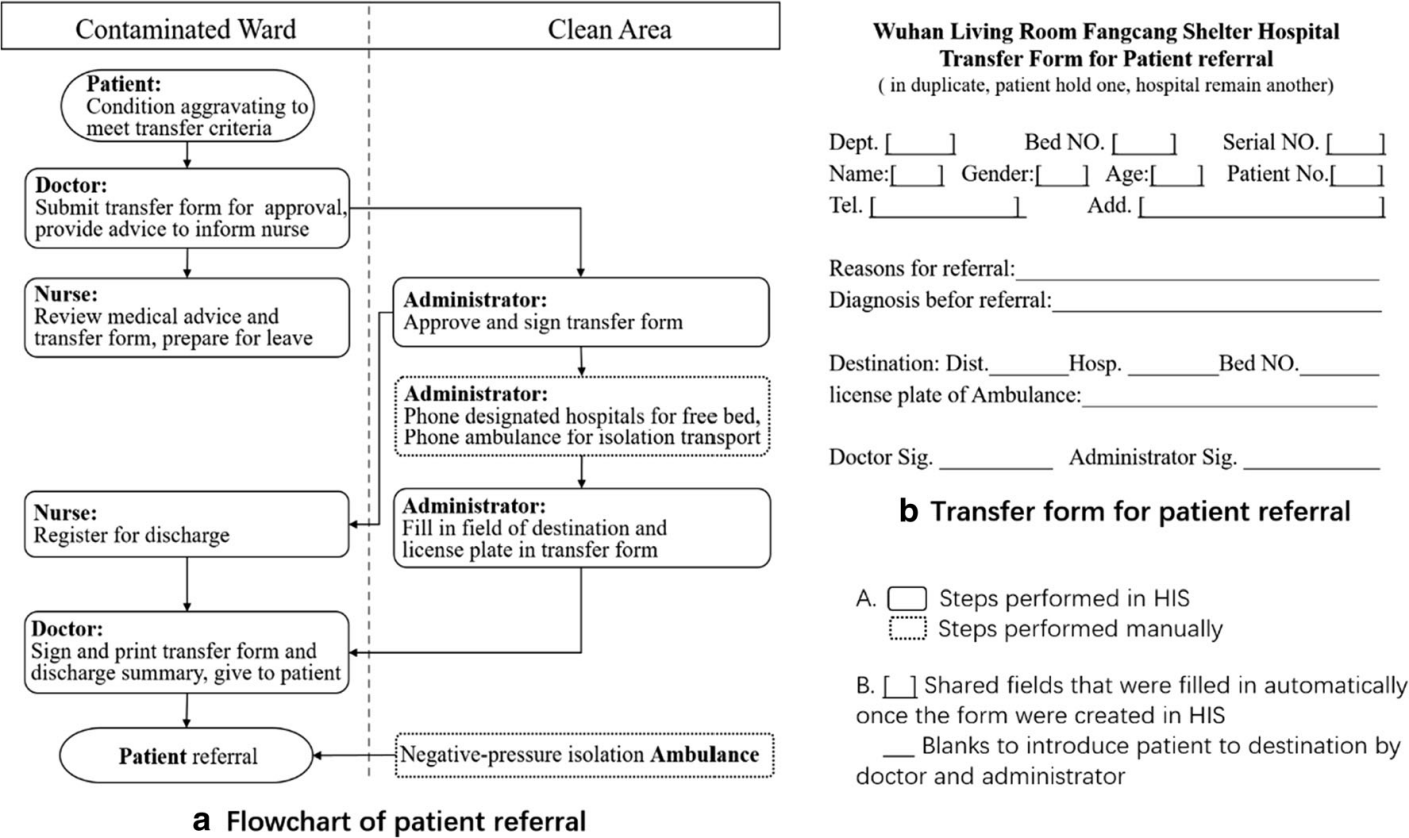
Flowchar and transfer from for paient referral in Wuhan Living Room Fangcang Shelter Hospital

Patient referral in Fangcang shelter hospital, whether to a designated hospital or a community isolation site, also followed a specific procedure. The flowchart of referral is illustrated in Fig. 1A. The procedure was initiated by the attending doctor and nurse in charge of the patient and the medical administration department of Fangcang shelter. All commutation among participators involving in referral depended on patient referral modules in HIS. First, the attending doctor decided whether the patient met transfer criteria. If they met, the doctor would ask for an administrator’s approval through a transfer form in HIS (Fig. 1B), and order the nurse in charge to prepare. The nurse reviewed the medical order and transfer form, registered for discharge, and prepared for the patient’s leave. Meanwhile, an administrator received and approved the transfer form and phoned designated hospitals or community isolation sites for help. Once a hospital or an isolation site replied, which meant an appropriate bed and doctor were available for the patient, the administrator would add the destination in the transfer form and send it back to the doctor’s and nurse’s workstation interface. Then, the complete transfer form and discharge summary would be printed from HIS, signed by doctor, and carried by the patient himself. Subsequently, the administrator called ambulance to transport the patient to the receiving place.

### Medical record and Mobile system

Medical records in Fangcang hospital complied strictly with requirement for completeness and timeliness as in ordinary hospitals. The difference was that, since all the patients had the same disease, most of the medical documents were formatted, such as admission notes, progress notes, discharge summaries and diagnosis certificates. The formatted documents in HIS were consistent with medical norms and the templates were well-designed. Necessary items were listed in rows. Some of the items were automatically filled with values from shareable fields elsewhere. Other blanks required a clinician to fill them in. Since paper might serve as a vehicle for viral transmission, a patient’s medical record was not printed after his discharge. Instead, the records would be printed, disinfected and filed away a few days before the Fangcang shelter hospital suspended operation. Subsequently, the hospital would receive patients’ official request for the file and send it to them by express.

Except for the electronic medical records module in the desktop HIS, a mobile electronic medical record system was an alternative for doctors in Fangcang hospital. Doctors could log into the system on a mobile phone or PDA wherever the WLAN covered using a customized APP. They could look over existing comments and write new notes on the device. The system interface was user-friendly, since most pages consisted of forms and check boxes.

### Clinical type and statistical report

COVID-19 patients were divided into four clinical types: mild cases, moderate cases, severe cases and critical cases, according to the Diagnosis and Treatment Protocol for COVID-19 (Trial Version 4 and later) issued by National Health Commission of China [15]. The four types were different from ordinary critical condition notices, because they not only outlined the patient’s condition, but also the direction of patient treatment, referral, research and administration. The IT Dept. introduced a set of medical order items, which were synonyms of the four clinical types. When a patient was admitted, HIS would request medical advice in clinical type via counsel broadcasted on the doctor’s workstation interface. The medical advice would remain valid until a new medical order was issued. During the period of validity, the medical advice was illustrated with specific background color on screen (green, yellow, orange, red) to inform everyone who had interest in the patient. All medical advice and corresponding start time and deadline were saved in a database.

A patient’s clinical type, as well as patient admittance and referral, human resource allocation and medical resource expenditure, were required to be collected and summarized in daily reports for the Wuhan Municipal Headquarters for COVID-19 Prevention and Control. But the massive scale, one of the most important characteristics of Fangcang hospital [7], made this information difficult to summarize at a glance. Thus, engineers made several statistical reports to help managers of the hospital and the Wuhan Municipal Headquarters see the whole picture and align resources. All periodical reports were directly extracted from HIS and automatically updated each day. Most statistical reports required by the government were made by the same programmer and same program statement as the host hospital, taking advantage of the same report layout and data structure between the two hospitals.

### Laboratory test, imaging examination and pharmacy service

COVID-19 RNA extraction in Wuhan Living Room Fangcang Shelter Hospital were done in a vehicle-mounted mobile biosafety level-3 laboratory (MBSL-3 Lab) by a well-trained diagnostic team dispatched from Beijing. The MBSL-3 Lab was equipped with PCR analysers and could release subjects immediately after the specimen analyses were completed [16]. However, there was no information system for the lab, and information dissemination between the lab and wards was semi-manual. Doctors gave medical advice in HIS for patients who needed tests one day in advance. Workers in the lab summarized an order list later in the same day according to records in HIS and allocated each ward a time window in the next day for samples to be delivered manually. According to the order list, nurses in each ward would collect throat swab samples at an opportune time and send them to the lab along with a sample list. Then docimasters performed laboratory testing and input results into HIS after determining whether the results were positive or negative. Thus doctors, nurses and patients were informed and could print test report from HIS if necessary.

There were two sets of Computed Tomography (CT) in the Wuhan Living Room Fangcang Shelter Hospital. Both accessed the medical image teleconsultation system which had been built and run by Zhongnan Hospital of Wuhan University years ago. The radiographers could read and report subjects’ images both in Fangcang hospital and the host hospital. Doctors could also directly read the images and reports on HIS.

A temporary pharmacy was established to deal with drug affairs, including donation, storage and distribution. The pharmacy module in the host hospital’s HIS was adopted. Obvious change was not observed on working intreface in HIS for doctors and nurses, but was for pharmacists [17]. The biggest two differences was drug list and drug supply. A specialized drug list, which was different from the ordinary list, was adopted specially for COVID-19. This meant new medical order list, different usage notice, particular dispensing process in HIS. As for drug supply, there was litter work relating to drug purchase affair, but much relating to drug donation and drug allocation between Fangcang hospital, host hospital and the public. The information system had received well-directed optimization by pharmacists and engineers.

### Equipment maintenance and medical staff training

Equipment maintenance affairs of the IT Dept. in Fangcang shelter hospital included configuration and adjustment of HIS and improvement and repair of hardware. IT engineers took extra measures to do this work to avoid exposure to the coronavirus. First, a virtual cloud desktop system was deployed before equipment came into use, which gave control to all computers on LAN. Engineers could adjust settings of hardware, install application patches and distribute documents to workstations over the system in the clean area’s engine-room. Second, the IT Dept. was in contact with the isolation ward over intercom or telephone. If there was a problem about IT infrastructure, the end-user called a qualified engineer. The engineer could remotely connect to the terminal while in-call to examine and solve the problem. Third, for the maintenance of hardware devices, replacement was prioritized over repair. The IT Dept. of Fangcang hospital deliberately used a uniform type for each kind of device, such as computers, PDAs, printers, card readers, and so on. A surplus of devices of the same type, which were already installed, configured and upgraded, were prepared in storeroom outside of the isolation area. Once one device in use was broken, a substitute would be sent to the ward. Meanwhile, the broken one would be stored in a cubby-hole and ultimately subjected to strictly implemented steps for disinfection.

Medical workers in the Fangcang hospital were mobilized from many medical institutions in various cities and were rotated frequently to get enough rest. Informatization training was emphasized, and three steps were taken to help newcomers understand the workflow and application operation of HIS. First, the IT Dept. ran regular training courses for medical staff group by group before they checked into the isolation ward. For the account permission requests of HIS, the IT Dept. released approval process to the medical staff during training and correspondingly developed account management function module in HIS to deal with approval. Second, training documentation, including written materials, PowerPoint and video, was put on the desktop of doctors’ and nurses’ workstations. Third, the IT Dept. offered a 24/7 Q&A hotline to users. At least two engineers acted as operators to answer questions on the consultation hotline every day. Whenever and wherever users made a manual mistake while using the equipment, applied for authorization of HIS module or detected a software bug, the operators would immediately solve the problem via computer telecontrol. Based on the steps above, all medical workers had good command of IT infrastructure and reduced unwanted requests for help.

## Results

The Fangcang shelter hospital in Wuhan, Hubei province began to build at midnight of February 3rd. The Wuhan Living Room exhibition hall, which was about 125,000 square metres, was one of the first three sites for Fangcang shelter hospitals. Only two days later, it was turned into isolation wards with 2000 beds, 16 workstations for doctors and nurses, 2 pharmacies, and 1 entrance for patients. More than 1700 confirmed patients were treated in the hospital from 7th February to 8th March. Thousands of employees worked in the hospital. Medical assistance came from different hospitals in various provinces, such as Shanghai, Guangdong, Jiangsu, Ningxia, Xinjiang and Hubei. Non-medical staff, such as engineers, vigilantes and logisticians were called together from Wuhan. Owing to strict infection control measures, no medical staff or technical workers were infected with the virus during this period.

The IT Dept. in the Wuhan Living Room Fangcang Shelter Hospital was headed by a director with two groups and a staff of 10. These were skilled employees from the host hospital and worked on-site or from home. Engineers working on-site finished the deployment of network, servers and terminal devices within one day. Database, HIS and mobile electronic medical record system were completely installed and debugged half a day later. Function modules in HIS in Fangcang shelter hospital were modified by engineers working at home. Most of the modules, relating to patient admittance, referral, laboratory test, imaging examination, pharmacy service, clinical type and medical record, were finished at the same time as the first patient’s admittance. And templates and interfaces of medical records in desktop HIS and in the mobile electronic medical record system were redesigned once the Diagnosis and Treatment Protocol for COVID-19 was updated. Laboratory tests, imaging examinations and pharmacy services were effective and efficient in the dependent deployment strategy. The CT workload increased to 200 cases per day based on the medical image teleconsultation system, in contrast to 60 cases based on the self-contained report component. Dozens of statistical reports were developed for decision makers both in and out the hospital. For example, a report revealed patients who had signs of oxygen therapy based on respiratory rate and oxygen saturation in medical document. Thus, headquarters could learn who was waiting for respirator and referral and how many beds and medical personnel in designated hospitals should be prepared at any moment.

Almost 800 doctors, nurses and docimasters have registered at the Wuhan Living Room Fangcang Shelter Hospital and one thousand authorizations in HIS were provided for them. Authorization service in batch mode was provided during the training. Software engineers hosted about 15 times on-site training courses to inform the medical staff how HIS worked after their registration date. Meanwhile, the scattered authorization requests would be handled by engineers remotely from home. Equipment maintenance affairs kept running smoothly. Several devices were replaced and repaired during the period when the hospital was running, but no engineer ever entered the contaminated areas.

## Discussion and conclusion

The COVID-19 has been attacking hundreds of countries and thousands of cities. Since the start of the pandemic, there have been 88 million reported COVID-19 cases and over 1.9 million deaths as of 10 January 2021 [18]. In the past week, cases have increased faster globally for more areas heading into winter again, and different variants of SARS-CoV-2 [19, 20]. Governments and citizens around the world are going all-out to fight the epidemic. Most regions have enacted personal protective strategies and public health policie to roll back the rising trend of coronavirus infections, and provided medical treatment service to heal the sick. Faced with the rapidly increasing number of infections, medical systems are facing collapse in many countries, although more and more medical institutions were transformed into designated hospitals. The Fangcang shelter hospital, which acted both as public health policy and medical treatment service, may be exactly the alternative health authorities’ mind scrabbled for. Several countries have built Fangcang shelter hospital, with similar or different terminologies, such as Community Care Facilities in Singapore [21], Nightingale Hospitals in the United Kingdom [22], and field hospitals in the United States of Ameria [23, 24]. They received and cared confirmed cases during the first wave of epidemic in 2020, thus substantially blocked community transmission [25, 26] and rapidly eased pressure on local health care infrastructure [8, 27].

The Fangcang shelter hospital was proposed as a novel concept for responding to the public health emergency. It was created as a quick-response mechanism. Nosocomial infection control was given primacy in all workflows during the COVID-19 epidemic everywhere. What the IT Dept. of Fangcang shelter hospital did was in compliance with the two characteristics above. To avoid risk any contagion, the IT infrastructure was necessarily prepared before the first patient’s admission, which meant the IT Dept. was obliged to finish deployment within 48 h. The IT Dept. chose a dependent deployment strategy, allowing skilled hardware engineers and software engineers to get the job done swiftly and effectively. Hardware installation, application development and modification were done in parallel by two groups to save time. When patients arrived, the area around the stadium was divided into three parts: the contaminated ward, a semi-clean corridor and a clean area. The work area of the IT Dept. was confined to the clean zone. Owing to the person-to-person hotline, the virtual cloud desktop system and remote-control technology, workstations in wards could have problems solved quickly and directly. Software development, debugging and integration were done on a remote computer that was in control of the local computer. Meanwhile, in the interest of speed and safety, replacement was prioritized over repair. Equipment taken from the contaminated ward would be disinfected before reuse.

The scheme above has successfully supported the newly-built hospital running on IT infrastructure. There were no major issues with IT and none of the IT staff was infected. Meanwhile, there were several alternatives that were recommended to improve informatization and prevention levels in the Fangcang shelter hospital for anyone who follows. First, the cable network within the stadium may switch to wireless networks against the background of mature commercial use of 5G. A 5G + VPN all-wireless solution was put into use in another Fangcang shelter hospital in Wuhan [28]. Terminal computers in the hospital were divided into several network units that were covered by branch 5G wireless routers. The 5G wireless router was configured to access intranet via 5G signal in the Fangcang shelter hospital and was provided with 5G data to access the internet wirelessly. These were independently connected to the host hospital over a VPN tunnel built on the 5G wireless network. This setup was reported to be zero maintenance of network and allowed for efficient troubleshooting. Second, more mobile devices may raise work efficiency and protection level. Wide use of mobile applications for doctors and nurses were recommend and welcome. Compared to routine doctors’ and nurses’ desktop workstations in shared offices and bedside caregivers for every patient, the use of mobile device reduced movement of healthy medical staff among diagnosed and potentially infectious patients. Also, self-help or wearable health devices for patients that monitor temperature, oxygen saturation, and so on, would help to provide continuous condition assessment and timely interventions in Fangcang hospital. For material supply from outside to inside, robot grocery deliveries were suitable. Medical supplies and daily necessities would be transferred to a robot in the clean area and transported to receiver in contaminated area. When the robot returned, it would be disinfected by ultraviolet lights in the semi-clean area and kept uninfected in the clean area. Thus, nobody would be exposed to risk of infection. Third, there were two popular work-styles in China during the epidemic that were suggested for the Fangcang hospital. One was cloud-based services. The hospital may rent computing resource from a cloud service provider, such as Amazon Web Services and Alibaba Cloud, and deploy the applications on cloud. The cloud-based service matched the independent deployment strategy of IT infrastructure very well. It would improve the overall speed for informationization construction, eliminating the risk of infection for deployer and maintainer. With the application of a cloud-storage platform to the construction of a hospital picture archiving and communication system (PACS) storage system, the efficient storage and retrieval of the hospital images was achieved. Another suggested work-style was teleworking [29]. Remote medical consultation and tele-diagnosis of images, for example, were practical ways to dedicate resources outside the affected areas to fight against the coronavirus. Remote video conferencing was also popular for meeting social distancing guidelines. The IT Dept. should make adequate preparations for the demand for telecommuting.

Medical environments and technical support work have undergone a drastic change during the COVID-19 epidemic. Health workers should tailor the design of such prevention and control strategies to the novel disease. A well-designed informationization support scheme was critical to respond to key characteristics and essential functions of hospital against the disease. This practice should protect the medical staff, engineers and all relevant personnel from infection.

## Declarations

### Availability of data and material

There was no data or material to report for this study.

### Code availability

There was no code to report for this study.
